# Forty years of successful assisted reproductive technologies in Croatia

**DOI:** 10.3325/cmj.2023.64.377

**Published:** 2023-12

**Authors:** Velimir Šimunić, University of Zagreb, School of Medicine, Ret, velimir.simunic, gmail.com

## Decline of fertility is a major challenge in reproductive medicine

Infertility is a growing problem for humanity. The World Health Organization (WHO) defines infertility as a disease; thereby, an obligation is imposed to support and treat couples. Forty to fifty years ago, the incidence of infertility was 5%-6%, while the rate today is 18%. Infertility affects both women and men: female-factor infertility accounts for 30% of the cases, male-factor for 30%, and combined causes account for 20% of the cases. In the remaining 20%, the causes are unknown, and this type of infertility is declared idiopathic (Box 1). Due to such progression of infertility disorders, the WHO predicts that in 10 years as many as 30% of couples will have serious difficulties with conception. Infertility in humans is treated by experts in reproductive medicine, a growing discipline that focuses on human reproduction, research, and infertility treatment (1-3).

Box 1 **Causes of infertility in women and men**

**Table:** 

**Infertility in women**	**Infertility in men**
Damage to the fallopian tubes Anovulations Polycystic ovary syndrome Endometriosis Uterine abnormalities Pelvic inflammatory disorders Consequence of surgical procedures	Oligoasthenozoospremia* • low sperm count • reduced sperm motility • low viability • reduced morphology Azoospermia • secretive • obstructive Genetic disorders Inflammatory disorders

*In addition, there are more severe types of male infertility: cryptozoospermia and globozoospermia.

Therefore, *in vitro* fertilization (IVF) should be regarded as one of the ten greatest medical achievements of the last century. Thousands of scientific studies have been published on assisted reproductive technologies (ART) and procedures, offering new insights into the beginning of life, human reproduction, menstrual cycle endocrinology, and early pregnancy. These studies represent the collective success of physiology, embryology, and clinical reproductive medicine. In the 45 years of ART, there has been great progress and adoption of new technologies that have contributed to successful treatment of 70%-80% of infertility causes. To date, these methods have resulted in the births of 10 million healthy children worldwide, a figure that makes an important contribution of 2%-7% to the birth rate of many countries. With modern ART technologies, around 3.5 million IVF procedures are performed annually in 100 countries, with more than 3000 IVF centers and up to 800 thousand births (4) (Box 2). Despite many critics and opponents of medical procedures that interfere with the beginning of a person's life, IVF will remain the main type of infertility treatment until a more successful, safer, and universally acceptable therapy is found. Unfortunately, many other social activities have not shown advances in this regard.

Box 2 **State-of-the-art assisted reproductive technologies**

**Table:** 

**Assisted reproductive technologies**
*In vitro* fertilization
Intracytoplasmic sperm injection
Embryo transfer
*In vitro* maturation
Freezing - vitrification and cryopreservation of: • oocytes • sperm • ovarian/testicular tissue
Sperm collection by surgical procedures from: • testis • epididymis
Freeze-all technology
Fertility preservation • in women and men • elective • medical • oncofertility
Preimplantation genetic testing • screening for aneuploidies and monogenetic diseases
Gamete donation
Uterus substitution/surrogacy • transplantation of uterus • surrogate motherhood

Several facts about reproductive aging have not been fully appreciated so far. Along with population aging, in the last 50 years human fertility has been strongly affected by changing life circumstances. Life-style (nutrition, sedentary life) and environmental toxicity (toxic pollutants), especially in industrial societies, are associated with a reduced reproductive potential of both women and men.

**Figure 1:**
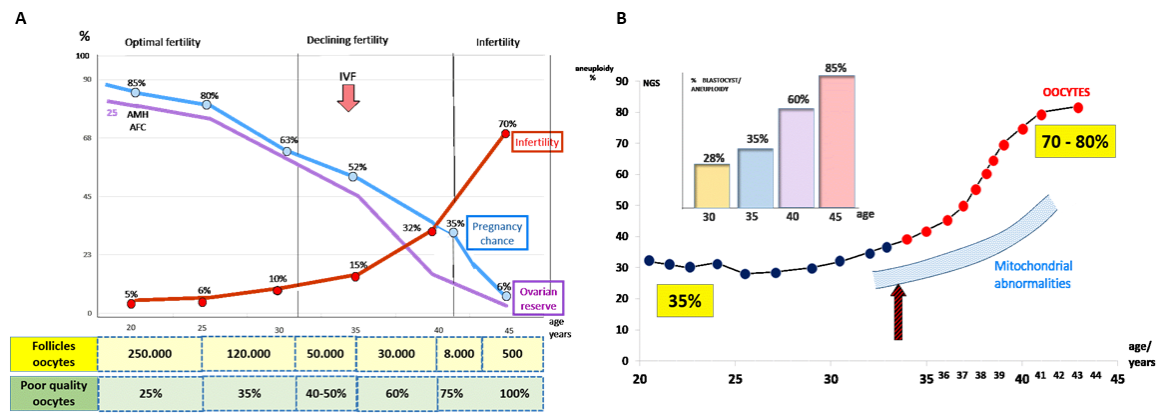
Decline of reproductive potential in women with aging (**A**), accompanied by increased aneuploidy rate and mitochondrial abnormalities in oocytes (**B**). AMH - antimüllerian hormone; AFC - antral follicular count; IVF - *in vitro* fertilization; NGS - next generation sequencing.

A woman is most fertile between the ages of 22 and 25, when fecundability (achieving pregnancy in 1 cycle) is 30%-35%. At the age of 30, it is already reduced to 20%. A woman is born with a finite number of oocytes, which are constantly consumed and cannot be renewed. Of the 400 000 oocytes with which a woman enters puberty, through the complex mechanisms of selection of a healthy oocyte destined for fertilization, 400 ovulations occur in her life, while the follicles recruited for the cycle are degraded (4). Due to the rapid reduction of oocytes, only 30 000 remain after the age of 35, and 10 000 after the age of 40. With aging, the proportion of chromosomally abnormal oocytes increases due to continuous selection of healthy oocytes. Already from the age of 35, the proportion of aneuploid oocytes, as well as mitochondrial abnormalities, increases significantly. After fertilization, the first mitoses and activation of the embryonic genome can increase mosaic errors. From the age of 42, we record 80% of aneuploidies, which are the main reason for the low probability of an orderly pregnancy (Figure 1). Today, even with IVF, no treatment can restore the intrinsic competence of gametes and embryos or affect the reduction of the ovarian reserve and the incompetence of oocytes. This is the reason why the age of over 35 is considered as an advanced maternal age. The reduced reproductive potential of women with advanced maternal age is characterized by a low ovarian reserve (increased follitropin, low antimüllerian hormone, and antral follicular count), and reduced embryonic development rate. The described findings reduce the possibility of a live birth with IVF treatment (4).

There is also an ongoing scientific debate about the constant decline in male fertility. In the last 50 years throughout the industrialized world, male infertility increased and spermatogenesis in fertile and infertile men decreased by 50%. A recently published meta-analysis by Levin et al, encompassing 223 studies of unselected men from all continents, found a 51.6% reduction in sperm concentration, count, and motility from 1973 till present (5). Since 2000, the number of sperm per ejaculate has declined by 2.64% per year, which is twice as fast as before. Such a rapid drop in sperm production (from 101 million/mL to 49 million/mL) has no genetic basis and is probably a result of environmental toxicity. Furthermore, testosterone production also declined, probably due to the combined effects of obesity and synthetic estrogens in the environment. Science tries to relate these findings to the effects on reproduction, but also to the growing incidence of testicular cancer. Other potential disruptors are smoking, alcohol, drugs, and physical and emotional stress. Oxidative stress is certainly a factor that negatively affects all stages of spermatogenesis (6). In recent years, the connection between the use of mobile phones and a reduced seed quality has been increasingly emphasized. Mobile phones emit electromagnetic waves that can be absorbed by the body. Experimental and observational studies have proven that radiofrequency electromagnetic fields can negatively affect gametes, spermatogenesis, testes, DNA fragmentation, sperm motility, morphology, and viability (7). Here, we have to take into consideration that humans have significantly lower fertility than other species, and this low rate is further deteriorated by age and unhealthy lifestyle. There still prevails an incorrect opinion that IVF can compensate for the natural, physiological decline in fertility due to aging, primarily in women but also in men.

## Croatia - the seventh country in the world to introduce IVF

The progress of IVF in the world has been slow, painstaking, and debatable. When Robert Edwards successfully fertilized a human egg outside the body in 1969, an angry mob set out to destroy his laboratory in Cambridge and was fortunately stopped in that intention. After numerous other studies, and about a hundred unsuccessful IVF attempts, an orderly IVF pregnancy resulted in the birth of Louise Brown in 1978. The cry of the first child conceived outside the mother's body was heard by the whole world, and the researchers responsible for this success, Robert Edwards and Patrick Steptoe, gained international fame and reputation. In 1980, successful IVF procedure was completed in Australia (Ian Johnston and Alex Lopata), and in 1981 in the USA (Georgina and Howard Jones). In 1982, Germany, Austria, and France followed suit, and in 1983 three more countries joined this elite club - Belgium, Canada, and Croatia. To be the seventh country in the world to achieve successful pregnancy and delivery via IVF was considered a sensational success for Croatia, and significantly strengthened the reputation of the Clinic for Women's Diseases and Childbirth, University Hospital Center Zagreb (popularly known as the Petrova Clinic according to the street where it is located).

**Figure 2:**
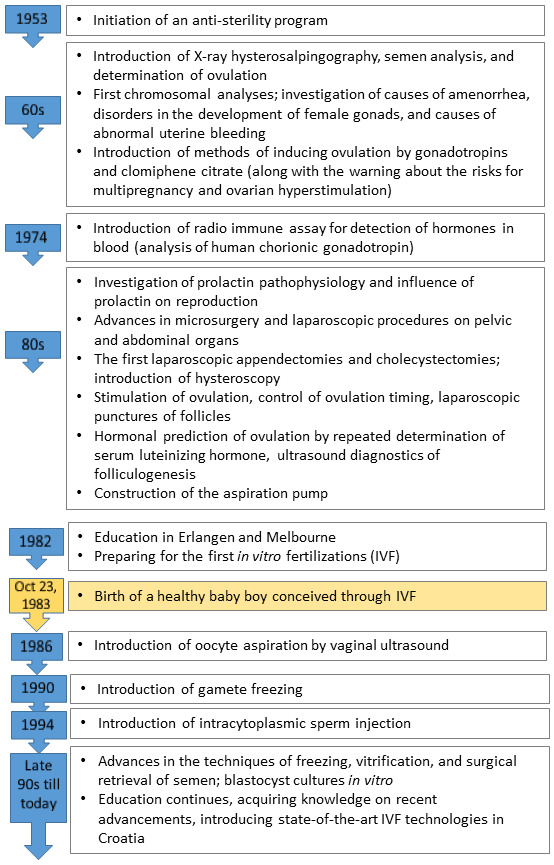
Seventy-year tradition of success in the treatment of infertility and gynecological endocrinology in Croatia.

As everywhere, the beginnings of IVF in Croatia were uncertain, complex, and difficult. Infertility treatment and gynecological endocrinology in Croatia have a rich, 70-year tradition (Figure 2). In 1953, an anti-sterility program was initiated at the Clinic for Women's Diseases and Childbirth in Petrova. Over the next two decades, professors Predrag Drobnjak, Veselko Grizelj, and Ivo Puharić were the bearers of progress in this discipline. Further contribution to the progress was certainly made by the top endocrinology laboratory led first by Professor Neda Longhino and later by Professor Ernest Suchanek. In the beginning, the possibilities of anti-sterility procedures were very modest, and the predominant treatment challenge were consequences of inflammation, including genital tuberculosis. Since then, the world’s progress in gynecological endocrinology and reproductive medicine has been quickly transferred to the Clinic in Petrova, which has remained the leading center for reproductive medicine in the region.

The birth of Louise Brown impressed the doctors of the Institute for Human Reproduction in Petrova, and strengthened the desire of younger experts to adopt this advancement of reproductive medicine. However, this intention was not supported by older physicians, who believed that the scope of the activity at that time was optimal for the Clinic. Without material help, expensive equipment, and with numerous obscurities and unknowns in the process, there was skepticism about the final success. At that time, each laboratory created a recipe for the embryo feeder, and the details of the procedure were kept secret. The success rate of treatment was only 5%-7% and there were few centers with proven success. Knowledge was reluctantly transferred, education was often expensive, and waiting lists for training in the area were long.

However, we were full of enthusiasm and self-confidence. Also, fortunate circumstances helped us in our efforts. Embryologists Tanja and Fredi Kniewald from Zagreb, who participated in the German IVF program (Erlangen), held an affirmative lecture in Zagreb. This dispelled our doubts, and in the fall of 1982, we began training with leading experts, Professor Trotnow and Engineer Kniewald in Erlangen and with Professor Alex Lopata in Melbourne. We are still grateful to our IVF teachers, without whom we probably would not have succeeded, or the results would have been significantly delayed.

At the end of 1982, we were finally preparing the first couples for IVF treatment. We decided on the method using stimulated cycles; laparoscopic oocyte aspiration was performed based on the calculation of the ovulatory luteinizing hormone surge. Surprisingly, our second embryo transfer (performed by Dr Šimunić, Engr. Suchanek, and Engr. Maćaš) resulted in a normal pregnancy. On Sunday, October 23, 1983, labor guided by Dr Šimunić began late in the evening, and a healthy baby boy was born by a cesarean section! Together we celebrated the sensational success of Zagreb and Croatian medicine. Already the same night, the news spread all over the country and beyond (8).

In the first six months after introducing IVF, we achieved five clinical pregnancies, with a success rate of 6%. This was surely an unexpected success in the age of many improvisations, transfer of embryos in a thermos, abdominal ultrasound, and only one catheter for embryo transfer. Since then, all subspecialists and embryologists have been committed to helping infertile couples achieve parenthood. Children born through IVF are healthy and show normal psychophysical development. There are slight risks of abnormalities, attributable to the age of the treated, the causes of infertility, and the choice of an inappropriate technology. Risks are also conferred by recognized or unrecognized comorbidities, the main ones being multiple pregnancies and prematurity (9).

The newly achieved fame of the Institute for Reproductive Medicine in the Petrova Clinic led to an influx of patients from all over the former country, but also from foreign countries, especially the Eastern Bloc. We also made a permanent effort to transfer knowledge and experience to experts from the region and friendly countries. The workload was high, and due to the natural timing of ovulation, laparoscopic oocyte aspirations were often performed at night, every weekend. In the first years of IVF treatment, we performed more than a thousand laparoscopies, a third at night. At that time, no material rewards or days off were offered in compensation.

From the initial modest results of IVF, the success rate gradually increased compared with other centers. Experts and management of our Clinic established strict ethical principles for all IVF procedures. We have been adopting new procedures and advanced technologies from other centers relatively quickly, with a delay of one to two years, regardless of weaker material possibilities. With such progress, the Petrova Clinic remains among the world’s top institutions regarding the scope and results of IVF. In Croatia, the entire ART program is carried out except for preimplantation genetic testing, donation, and surrogacy, which is illegal in Croatia. Because of ART availability in Croatia there is no need for treatment abroad, which is not more successful but is significantly more expensive. According to the Global Clinic Rating, we are still among the first 20 or so countries in terms of success. Today, in 16 IVF centers in Croatia, 6000-7000 procedures of medically assisted fertilization are performed annually, and 1600-1800 children are born. This an impressive 5% contribution to the decreased birth rate in Croatia. From the initial 5%, the overall success rate in recent years has reached 30%. In the 40 years of treatment with ART, 40 000 children have been born in Croatia, and about 18 000 in our Clinic. Only in the last seven years, 12 000 children have been born in Croatia thanks to medically assisted fertilization treatment. Such a contribution to Croatian reproductive medicine was made possible by the pioneers and giants of this field, but also by their students who followed in their footsteps, especially embryologists, technicians, and specialized nurses.

The Institute for Human Reproduction of the University Hospital Center Zagreb has been the main and most successful center for reproductive medicine in the country and the wider region. It is also an educational center and knowledge incubator for young professionals. We also need to acknowledge the significant contribution of private IVF centers. Our reward for effort and enthusiasm is the great privilege to pass on knowledge to younger generations. It gives us huge satisfaction to participate in the realization of the fundamental human right to create a family and have reproductive autonomy. As physicians, we are especially grateful for the honor that through our work we helped people fulfil their greatest wish – to become parents.
